# Association of frontal fibrosing alopecia and contact allergens in everyday skincare products in Hispanic females: a case-control study^☆^^☆☆^

**DOI:** 10.1016/j.abd.2020.09.013

**Published:** 2021-09-24

**Authors:** Sonia Sofia Ocampo-Garza, Maira Elizabeth Herz-Ruelas, Sonia Chavez-Alvarez, David Marcelo de la Fuente-Rodriguez, Jorge Ocampo-Candiani

**Affiliations:** a Dermatology Department, University Hospital “Dr. José Eleuterio González”, Universidad Autónoma de Nuevo León, Monterrey, Mexico; b School of Medicine, Universidad Autónoma de Nuevo León, Monterrey, Mexico

Dear Editor,

Frontal Fibrosing Alopecia (FFA) is primary lymphocytic scarring alopecia with an unclear etiopathogenesis. Hormonal, immune-mediated, genetic, and environmental hypotheses have been proposed; one of which relates to increased sunscreen use.^1^ We sought to identify a possible association between FFA and sunscreen use in our patient population.

A case-control study with thirty-six Hispanic females; eighteen cases and eighteen sex-and age-matched controls, was designed. The protocol was approved by the local ethics committee of the University Hospital “Dr. José Eleuterio González” in Monterrey, Mexico. A questionnaire inquiring about hygiene products and sunscreens, including time of use, was formulated. Patients and controls were patch tested using allergens from allergEAZE’s Standard, Cosmetic, and Photopatch series. Readings were performed 48 and 96 hours after application. Reactions were measured by the North American Contact Dermatitis Group criteria.^2^ FFA patients seen in our Dermatology clinic from 2012–2018 were included. Fisher’s exact test was used to compare between groups. Statistical significance was considered p < 0.05.

The mean age of FFA patients was 59.1 and 56.5 years for controls. Sixteen cases were postmenopausal. Ten subjects referred minimally one symptom (mostly pruritus). Seven cases had at least one autoimmune-mediated disease, being lichen planus pigments the most common. Nine patients had a family history of autoimmunity.

Hair loss outside of the scalp was noted in fifteen cases. All had eyebrow, twelve limb, and ten eyelash involvements. Eight individuals presented facial papules.

Table 1 outlines the use of hygiene products. Sunscreen use in FFA subjects was almost double in comparison with healthy controls (**p = 0.035**). Makeup foundation was used by fourteen patients in each group. FFA cases used more facial moisturizers compared to controls, without statistical significance.

**Table 1:** Personal hygiene products used by cases and controls.

Product	FFA, n (%)	Controls, n (%)	p-value
Makeup foundation	14 (77.8)	14 (77.8)	0.999
Sunscreen	15 (83.3)	8 (44.4%)	**0.035**
Makeup remover	10 (55.6)	11 (61.1)	0.999
Facial moisturizer	17 (94.4)	14 (77.8)	0.338
Anti-aging cream	12 (66.7)	6 (33.3)	0.094
Shampoo	18 (100)	18 (100)	N/A
Conditioner	9 (50)	12 (66.7)	0.5
Hair styling cream	8 (44.4)	9 (50)	0.999
Hair treatments	4 (22.2)	4 (22.2)	0.999
Hair dye	11 (61.1)	14 (77.8)	0.471
Permanents (wave or hair straightening)	1 (5.6)	3 (16.7)	0.603
Deodorant	15 (83.3)	18 (100)	0.229
Perfume	14 (77.8)	16 (88.9)	0.658

Fifteen patients had positive reactions to at least one allergen against ten controls, no significant difference was found. The most common allergens in FFA were iodopropynyl butylcarbamate and propolis (Table 2). We reviewed patients’ hygiene products and made avoidance recommendations.

**Table 2:** Most common positive contact allergens.

Allergen	FFA, n (%)	Controls, n (%)	p-value
Amerchol L101	0 (0)	2 (11.1)	0.486
Fragrance mix 8%	0 (0)	2 (11.1)	0.486
Iodopropynyl butylcarbamate	3 (16.7)	1 (5.6)	0 603
Nickel sulfate hexahydrate	1 (5.6)	2 (11.1)	0.999
Propolis	3 (16.7)	1 (5.6)	0.603
Methyldibromo glutaronitrile	2 (11.1)	2 (11.1)	0.999
Sodium disulfite	0 (0)	2 (11.1)	0.486

Aldoori et al. suggested environmental factors associated to FFA; with a significant difference in sunscreen use compared to controls. Forty patients were patch tested; more positive reactions to linalool hydroperoxide and Balsam of Peru were found in subjects with FFA versus general patch tested subjects.^3^ Rocha et al. did not observe differences in sixty-three FFA patients in comparison with local patch tested individuals, nor photoallergy was detected.^4^ Identified allergens in FFA are unable to explain the association between disease and sunscreen use.^5^ Thompson et al. found Titanium Dioxide (TiO_2_) nanoparticles in hair shafts of FFA patients and asymptomatic controls; no positive reactions in eight FFA patients patch tested to five titanium types were found. It is unclear if TiO_2_ plays a role in FFA pathogenesis.^6^ Recently Rudnicka et al. patch tested 20 patients with FFA and 24 healthy controls finding a positive patch test reaction to one or more allergen in 65% of the cases, while only 37.5% of the controls had a positive reaction. The most common allergens encountered in the FFA group were cobalt chloride hexahydrate 35%, nickel sulfate hexahydrate 25%, and potassium dichromate 15%.^7^ In our study, 83.3% of cases and 55.5% of controls had at least one positive allergen.

Our study included sex-and age-matched healthy controls, while most of the previous publications have a general patch test population. Limitations include small sample size, recall bias for sunscreen use, lack of genetic studies, and generalizability due to racial/ethnic homogeneity.

In conclusion, FFA’s etiopathogenesis remains unknown. Increased sunscreen use and a greater prevalence of contact allergy was demonstrated in our FFA patients. Raising the question of whether allergic/immunologic responses in genetically predisposed patients could be triggered by exogenous substances. Our statistically non-significant greater prevalence of contact allergy and heterogeneity of results in previous studies endorses the need for further research to confirm this association.

## Financial support

None declared.

## Authors' contributions

Sonia Sofia Ocampo-Garza: Conception and design of the study; acquisition of data; analysis and interpretation; drafting the article; final approval of the version submitted.

Maira Elizabeth Herz-Ruelas: Conception and design of the study; analysis and interpretation; revising the article; final approval of the version submitted.

Sonia Chavez-Alvarez: Conception and design of the study; revising the article; final approval of the version submitted.

David Marcelo de la Fuente-Rodriguez: Acquisition of data; final approval of the version submitted.

Jorge Ocampo-Candiani: Conception and design of the study; analysis and interpretation; revising the article; final approval of the version submitted.

## Conflicts of interest

None declared.
